# Structural requirement of Ntc77 for spliceosome activation and first catalytic step

**DOI:** 10.1093/nar/gku914

**Published:** 2014-10-07

**Authors:** Hsin-Chou Chen, Kae-Jiun Chang, Yu-Lun Su, Yu-Hsin Huang, Soo-Chen Cheng

**Affiliations:** 1 Institute of Molecular Biology, Academia Sinica, Nankang, Taipei, Taiwan 115, Republic of China; 2 Institute of Microbiology and Immunology, National Yang-Ming University, Shih-Pai, Taipei, Taiwan 112, Republic of China

## Abstract

The Prp19-associated complex is required for spliceosome activation by stabilizing the binding of U5 and U6 on the spliceosome after the release of U4. The complex comprises at least eight proteins, among which Ntc90 and Ntc77 contain multiple tetratricopeptide repeat (TPR) elements. We have previously shown that Ntc90 is not involved in spliceosome activation, but is required for the recruitment of essential first-step factor Yju2 to the spliceosome. We demonstrate here that Ntc77 has dual functions in both spliceosome activation and the first catalytic step in recruiting Yju2. We have identified an amino-terminal region of Ntc77, which encompasses the N-terminal domain and the first three TPR motifs, dispensable for spliceosome activation but required for stable interaction of Yju2 with the spliceosome. Deletion of this region had no severe effect on the integrity of the NTC, binding of NTC to the spliceosome or spliceosome activation, but impaired splicing and exhibited a dominant-negative growth phenotype. Our data reveal functional roles of Ntc77 in both spliceosome activation and the first catalytic step, and distinct structural domains of Ntc77 required for these two steps.

## INTRODUCTION

Introns are removed from pre-mRNA via two consecutive transesterification reactions catalyzed by the spliceosome. The spliceosome is composed of five small nuclear RNAs (snRNAs), U1, U2, U4, U5 and U6, in the form of small nuclear ribonucleoprotein particles (snRNPs), and numerous protein factors. Coordinated actions of these factors with serial structural changes of the spliceosome form the catalytically active spliceosome to allow the reactions to take place (for review, see 1). In brief, U1, U2 and the U4/U6.U5 tri-snRNP bind to pre-mRNA in a sequential manner to assemble the spliceosome. A subsequent conformational rearrangement results in the release of U1 and U4, mediated by DExD/H-box proteins Prp28 and Brr2, respectively (2–4). A protein complex associated with Prp19, called NTC (for nineteen complex), is then added to the spliceosome to stabilize the association of U5 and U6 with the spliceosome, and promote specific interactions of U5 and U6 with pre-mRNA (5,6).

In preparation for the first catalytic reaction, U2 snRNP subcomplexes SF3a and SF3b are destabilized from the spliceosome, mediated by DExD/H-box protein Prp2, presumably to free the branchpoint to allow the reaction to initiate (7,8). Two proteins, Yju2 and Cwc25, are then required to promote the first chemical reaction (8,9). Cwc25 binds to the spliceosome exclusively after the action of Prp2 and only in the presence of Yju2 (9), whereas Yju2 can be recruited to the spliceosome prior to or after the action of Prp2 via interactions with NTC components Ntc90 and Ntc77 (10). After the first reaction, Yju2 and Cwc25 are destabilized, mediated by another DExD/H-box protein Prp16 (11), to free the splice sites and allow the interactions of the 5′ and 3′ splice sites. Binding of Slu7, Prp18 and Prp22 promotes the second chemical reaction (12–14). The spliceosome utilizes the strategy of remodeling its structure to position and reposition the splice sites in facilitating the progression of the reaction during catalytic steps.

NTC has previously been demonstrated to mediate spliceosome activation by mediating specific interactions of U5 and U6 with pre-mRNA to stabilize their association with the spliceosome. In addition, NTC is also required for the release of Sm-like protein (Lsm) from binding to the 3′-end of U6 snRNA, which can then interact with the intron in a region ∼30 nt downstream from the 5′ splice site (6). Eight proteins have been identified to be core components of NTC, including essential splicing factors Prp19, Cef1/Ntc85, Clf1/Syf3/Ntc77 and Syf1/Ntc90, and non-essential splicing factors Snt309/Ntc25, Syf2/Ntc31, Isy1/Ntc30 and Ntc20 (15–19). They function as an integral complex and associate with the spliceosome simultaneously. Proteomic studies of proteins associated with Cef1/Ntc85 and its orthologs have identified similar protein complexes in the fission yeast and human, indicating evolutionary conservation of this complex (20,21). The Cef1/Ntc85-associated complex, named CWC for complexed with Cef1 in *Saccharomyces cerevisiae*, contains many other proteins in addition to those identified in NTC (21). Nevertheless, several of them were found to have distinct functions from NTC. Among them, Yju2/Cwc16, Cwc22 and Cwc25 are required only after spliceosome activation and function in the first catalytic step (9,10,22). Immunoprecipitation analysis also revealed that they are only loosely associated with NTC, and do not function as a complex (9,10,22). Several other proteins have also been suggested to be related to NTC (23).

Ntc77 was named Clf1 (crooked neck-like factor) for its homology to *Drosophila* crooked neck protein (24), and was also identified as Syf3 (synthetic lethal with *cdc* forty) in a genetic screen for synthetic lethality to *cdc40*/*prp17* (25). The Ntc77 protein is highly conserved throughout the evolutionary scale, and the sequence contains 15 tandem tetratricopeptide repeat (TPR) elements found in many protein complexes (26). The TPR motif is defined by a stretch of 34 amino acid residues, containing eight loosely conserved residues with conserved amino acid type and spacing, and each motif forms two anti-parallel α-helices (26). TPR elements are usually tandemly arrayed with more than three copies within a polypeptide, and one or two TPR-elements residing outside the TPR array are also normal (26). TPR-containing proteins were found in many protein complexes as a scaffold protein. It has been proposed that tandem TPR elements form a right-handed superhelical structure in which the groove is responsible for interaction with an α-helix of the target protein (27).

Ntc77 interacts with all components of NTC except Prp19 and Ntc25 (18). Another NTC component Ntc90 also contains multiple TPR elements, and can form a subcomplex with Ntc20, Ntc30 and Ntc31 (18). Ntc90 plays a role in the first catalytic reaction by recruiting Yju2 to the spliceosome, and is not required for spliceosome activation (28). Yju2 is composed of two distinct domains with a conserved N-terminal half (Yju2-N), which is partially functional by itself, and a less conserved C-terminal half (Yju2-C), which by itself has no function. Combination of Yju2-N and Yju2-C confers full function of Yju2 both *in vivo* and *in vitro* (29). Yju2-N interacts with Ntc90, and Yju2-C interacts with Ntc77. Despite not being functional, Yju2-C binds to the spliceosome in a stable manner, presumably via interaction with Ntc77, and the presence of Yju2-C stabilizes the association of Yju2-N with the spliceosome (29). It is therefore possible that Ntc77 also plays a role in the first catalytic reaction through its interaction with Yju2.

Here, we show that Ntc77 has dual functions in the spliceosome pathway, required for both spliceosome activation and the first chemical reaction. Depletion of Ntc77 disrupted the integrity of NTC, and prevented the binding of NTC components to the spliceosome. We have also identified an N-terminal region of Ntc77, comprising the N-terminal domain (NTD) and the first three TPR elements (N-3), important for stable association of Yju2 with the spliceosome. Deletion of the N-3 region did not affect the integrity of NTC, the binding of NTC to the spliceosome or spliceosome activation but destabilized the association of Yju2 with the spliceosome. As a result, cells with Ntc77 lacking the N-3 region exhibited dominant-negative growth phenotype with the spliceosome stalled at the pre-catalytic step.

## MATERIALS AND METHODS

### Yeast strains

Yeast strains used were BJ2168 (MAT**a** prc1 prb1 pep4 leu2 trp1 ura3), YSCC1 (MAT**a** prc1 prb1 pep4 leu2 trp1 ura3 PRP19-HA), YSCC12 (MAT**a** his3 his7 ade3 ura3 prp2-1 PRP19-HA), YSCC103 (MAT**a** prc1 prb1 pep4 leu2 trp1 ura3 PRP19-HA LSM3-V5), YSCC773 (MAT**a** prc1 prb1 pep4 leu2 trp1 ura3 URA3::GAL1-NTC77).

### Oligonucleotides

The following oligonucleotides were used:

77-4, GGCGAATTCGATCCAGC
77-5, GGCCGGATCCGAATTCATATGGACACTTTA
77-6, GGCCGGATCCATATGCCCAGAGTAGACAAG
77-7, GGCCGGATCCATATGGACGACATCATCCCTCA
77-8, CCGGAAGCTTAGTGAAATGTTTATGAGGG
77-9, CTCGCAACATCAGTTAC
77-10, GCGCAGATCTTAGTAGAGTACTTTTAGC
A1, CTTCATCACCAACGTAG
R13, GAGTGACGATTCCTATAG

### Antibodies and reagents

The anti-V5 antibody was purchased from Serotec Inc. The 8G5F antibody against hemagglutinin (HA) was produced by immunizing mice with a keyhole limpet hemocyanin-conjugated HA peptide (T.Y. Tsao and S.-C. Cheng, unpublished results). Antibodies against Prp19, Ntc90, Ntc85, Ntc77, Ntc30, Ntc25, Ntc20, Yju2 and Smd1 were produced by immunizing rabbits with corresponding recombinant proteins. Anti-Snu114 antibody was raised against amino acid residues 1–129 of Snu114 fused with GST, anti-Prp8 antibody against amino acid residues 1–115 of Prp8 and anti-Prp9 antibody against amino acid residues 1–161 of Prp9. RNasin and SP6 RNA polymerase were from Promega. Streptavidin Sepharose and protein A-Sepharose (PAS) were purchased from GE Healthcare Inc.

### Construction of *NTC77* deletion mutants

To generate plasmids for overexpression of Ntc77, a plasmid vector, pRS426ΔCHV.GAL, was first contructed by insertion of a 0.8-kb DNA fragment containing the *GAL1-GAL10* promoter region into *Eco*RI-*Bam*HI sites of pRS426 vector in which the enzyme sites from *Cla*I to *Eco*RV were deleted. For expression of wild-type Ntc77, a 2.8-kb DNA fragment containing the entire open reading frame (ORF) of *NTC77* with HA-tagged at the C-terminus and 0.7-kb of the downstream region was inserted into *Bam*HI-*Xba*I sites of pRS426ΔCHV.GAL vector to generate plasmid pRS426ΔCHV.GAL-NTC77-HA. For ΔN-3, a 0.9-kb *Bam*HI-*Bsu*36I DNA fragment generated by polymerase chain reaction (PCR) with oligonucleotides 77-6 and 77-4 was ligated to the *Bam*HI-*Bau*36I-cleaved pRS426ΔCHV.GAL-NTC77-HA. For Δ11–14, a 0.9-kb *Esp*I-*Hin*dIII DNA fragment generated by PCR with oligonucleotides 77-5 and 77-8 was ligated to the 8-kb *Esp*I-*Hin*dIII-cleaved pRS426ΔCHV.GAL-NTC77-HA. For Δ14-C, plasmid pRS426ΔCHV.GAL-NTC77-HA was cleaved with *Hin*dIII and *Cla*I, and then treated with Klenow fragment to repair the DNA ends for ligation. For ΔN-10, a 0.4-kb *Bam*HI-*Hin*dIII DNA fragment generated by PCR with oligonucleotides 77-7 and 77-4 was inserted into the *Bam*HI-*Hin*dIII-cleaved pRS426ΔCHV.GAL-NTC77-HA.

For expression of *NTC77* and its deletion variants under the control of its own promoter on single-copy plasmid, a plasmid vector, pRS414.GAL, was first contructed by insertion of a 0.8-kb DNA fragment containing the *GAL1-GAL10* promoter region into *Eco*RI-*Bam*HI sites of pRS414 vector. A 2.8-kb DNA fragment containing the entire ORF of *NTC77* with HA-tagged at the C-terminus and 0.7-kb of the downstream region was inserted into *Bam*HI-*Xba*I sites of pRS414.GAL vector to generate plasmid pRS414.GAL-NTC77-HA. A 1.35-kb *Kpn*I-*Bgl*II DNA fragment derived from a yeast genomic library clone was ligated with the 4.9-kb fragment of *Kpn*I-*Bgl*II-cleaved pRS414.*GAL-NTC77-HA* to generate plasmid pRS414.NTC77N. A 0.5-kb *Nco*I-*Bgl*II DNA fragment generated by PCR with oligonucleotides 77-9 and 77-10 was ligated with the 5.5-kb fragment of *Nco*I-*Bgl*II-cleaved pRS414.NTC77N to generate pRS414.P77. NTC77 and its deletion variants are cloned into pRS414.P77 by ligating the *Bam*HI-*Eag*I DNA fragments derived from the corresponding pRS426.ΔCHV.*GAL*-based plasmids with the 6-kb fragment of *Bgl*II-*Eag*I-cleaved pRS414.P77.

### Preparation of splicing substrates, extracts, NTC and NTC-related complexes

Substrates for the splicing reaction were generated by *in vitro* transcription using SP6 RNA polymerase. Plasmid pSP6Act88 was linearized with *Eco*RI for wild-type or *Cla*I for 3′-tail and exon 2-truncated transcript (Ac/Cla) as templates for the transcription reaction (6). Splicing extracts were prepared according to Cheng *et al.* (30). For preparation of *prp2* mutant extract, YSCC12 cells were grown at 25°C until mid-log phase, and then shifted to 37°C and grown for two more hours before harvest. For preparation of Ntc77-depleted extracts, YSCC773 cells cultured in uracil-dropout synthetic complete (SC) medium supplemented with galactose were inoculated into YPD and grown at 30°C for 12 or 16 h. For extracts prepared from YSCC773 harboring pRS414.NTC77-HA or pRS414.ΔN-3-HA, cells were grown in YPD for 20 h before harvest. NTC and its related complexes were purified according to the method described in Tarn *et al.* by chromatography on anti-HA antibody column (31).

### Immunodepletion, isolation of the spliceosome and spliceosome stability assays

For depletion of specific factors from yeast extracts, 25 mg of PAS was swollen in 1 ml of NET-2 buffer (50 mM Tris-HCl, pH 7.5, 150 mM NaCl, 0.05% NP-40) to make a bed volume of 100 μl, and was used for depletion of 200 μl extracts after conjugation of antibodies. For depletion of Cwc25, 200 μl of polyclonal anti-Cwc25 antibody was used. For depletion of Prp19 from Prp19-HA extracts, 200 μl of 8G5F antibody was used. For double depletion of Cwc25 and Prp19, 200 μl of anti-Cwc25 antibody and 200 μl of 8G5F antibody were conjugated simultaneously to 100 μl of PAS and used for depletion of 200 μl of extracts. Extracts were incubated with antibody-conjugated PAS at 4°C for 1 h, and supernatants were collected as depleted extracts. For precipitation of the spliceosome, antibodies were conjugated with 10 μl of PAS for precipitation of 10–20 μl of splicing reaction mixtures. For Ntc20, Prp8 or Smd1, 1 μl of polyclonal anti-Ntc20, anti-Prp8 or anti-Smd1 antibody was used. For V5-tagged Prp2 or Lsm3, 1 μl of anti-V5 antibody was used. Precipitation of the spliceosome with streptavidin Sepharose, spliceosome stability assays and ultraviolet (UV)-crosslinking analysis were performed as described in Chan *et al.* (6).

## RESULTS

### Ntc77 is required for formation of a functional form of NTC

**Figure 1.**
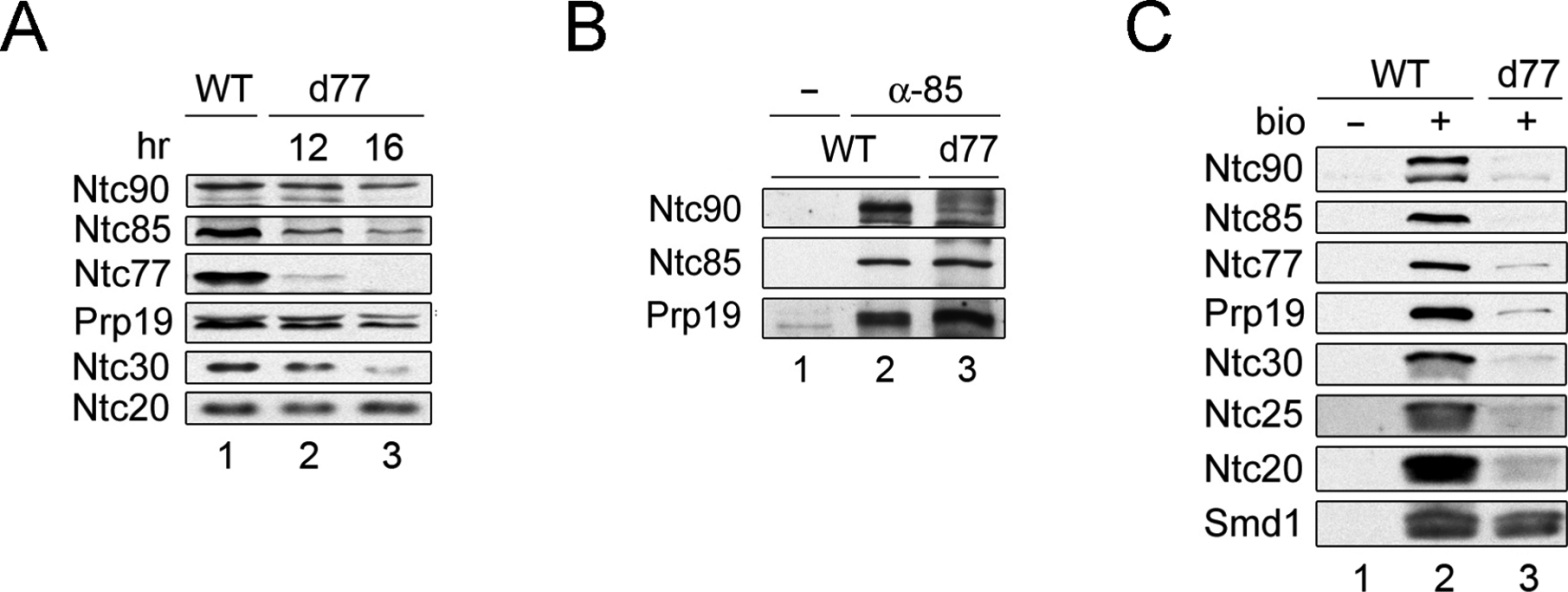
Ntc77 is required for the integrity of NTC and for its binding to the spliceosome. (**A**) Western blotting of total proteins of extracts prepared from wild-type (lane 1) or Ntc77 metabolically depleted cells after incubation in the glucose-based media for 12 (lane 2) or 16 h (lane 3). (**B**) Extracts prepared from wild-type (lanes 1 and 2) or Ntc77-depleted cells (16 h in glucose) (lane 3) were immunoprecipitated with no antibody (lane 1) or anti-Ntc85 antibody (lanes 2 and 3), and probed with antibodies against Ntc90, Ntc85 or Prp19. (**C**) Splicing reactions were carried out in wild-type (lanes 1 and 2) or Ntc77-depleted (12 h in glucose) (lane 3) extracts using non-biotinylated (lane 1) or biotinylated (lanes 2 and 3) ACAC pre-mRNA, and the spliceosomes were pulled down with streptavidin Sepharose. WT, wild-type; d77, Ntc77-depleted extracts.

Two components of NTC, Ntc90 and Ntc77, contain multiple copies of the TPR motif, which is implicated in protein–protein interactions (27). We have previously shown that Ntc90 is not required for spliceosome activation, and instead is essential for the recruitment of Yju2 for the first chemical reaction (28). To assess the function of Ntc77, we metabolically depleted Ntc77 using *GAL1*-promoter to test how formation of the NTC or the spliceosome was affected. Splicing extracts prepared from such cells after incubation in glucose-containing media for 12 or 16 h contained only residual amounts of Ntc77, while the amount of Ntc20 was not affected. The amounts of Prp19 and Ntc90 were slightly reduced, but that of Ntc85 and Ntc30 appeared to decrease more extensively after growth in glucose for 16 h (Figure 1A). The extract also contained residual amount of splicing activity, and could be rescued by the addition of purified NTC (Supplementary Figure S1).

We have previously shown that Prp19 forms a stable complex with Ntc25, which is important for stable interaction of Prp19 with Ntc85 (32). Ntc85 further interacts with Ntc77 and Ntc90 in formation of the NTC, and the interaction of Prp19 with Ntc85 is required for stable association of Ntc85 with Ntc77 and Ntc90 (18). We then tested whether Ntc77 is required for the interaction of Ntc85 with Prp19 or Ntc90. The splicing extract was subjected to immunoprecipitation with antibody against Ntc85 followed by western blotting to examine stable interaction of Ntc85 with Prp19 and Ntc90 in the absence of Ntc77. The result showed that while the interaction between Ntc85 and Prp19 was not affected in the absence of Ntc77, the interaction between Ntc90 and Ntc85 was compromised (Figure 1B). This is in contrast to depletion of Ntc90, which does not affect stable interaction of Ntc85 with Prp19 or Ntc77 (28). When Ntc90 is *in vivo* depleted, the amounts of Ntc30 and Ntc31 diminish, but the binding of the rest of the NTC components to the spliceosome is not affected (28). We then examined whether other NTC components could associate with the spliceosome in the absence of Ntc77. The spliceosome formed with biotinylated actin pre-mRNA was pulled down and the components were subjected to western blotting. The 3′ splice site mutant ACAC was used to avoid recycling of the spliceosome (33). The results showed that none of the NTC components could associate with the spliceosome in the absence of Ntc77 (Figure 1C, lane 3). Taken together, these results indicate that Ntc77 is required for formation of the functional form of NTC, and therefore for spliceosome activation.

### An Ntc77 domain dispensable for spliceosome activation

We have previously found that besides interacting with other NTC components, Ntc77 also interacts with first-step factor Yju2 (10). Yju2 is required for the first catalytic reaction after the action of Prp2, which promotes destabilization of SF3a/b, but can be recruited to the spliceosome prior to Prp2 action via interactions with NTC components Ntc90 and Ntc77 (10). The conserved N-terminal half of Yju2 interacts with Ntc90, binds to the spliceosome less tightly, and is partially functional. The less conserved C-terminal domain of Yju2 interacts with Ntc77, can bind to the spliceosome in a stable manner without promoting the first catalytic reaction and stabilize the interaction of Yju2-N with the spliceosome. The combination of the N- and C-domains assumes full Yju2 activity (29). The fact that Ntc77 interacts with Yju2-C, which stabilizes the association of Yju2-N with the spliceosome, indicates that Ntc77 might have functions in the first catalytic step in addition to its role in spliceosome activation. Based on these observations, we set out to dissect functional domains of Ntc77, predicting that deleting the domain required for the first catalytic reaction would result in retention of NTC on the spliceosome in a pre-catalytic but activated state, and cells overexpressing such deletion mutants would exhibit dominant-negative phenotype.

**Figure 2.**
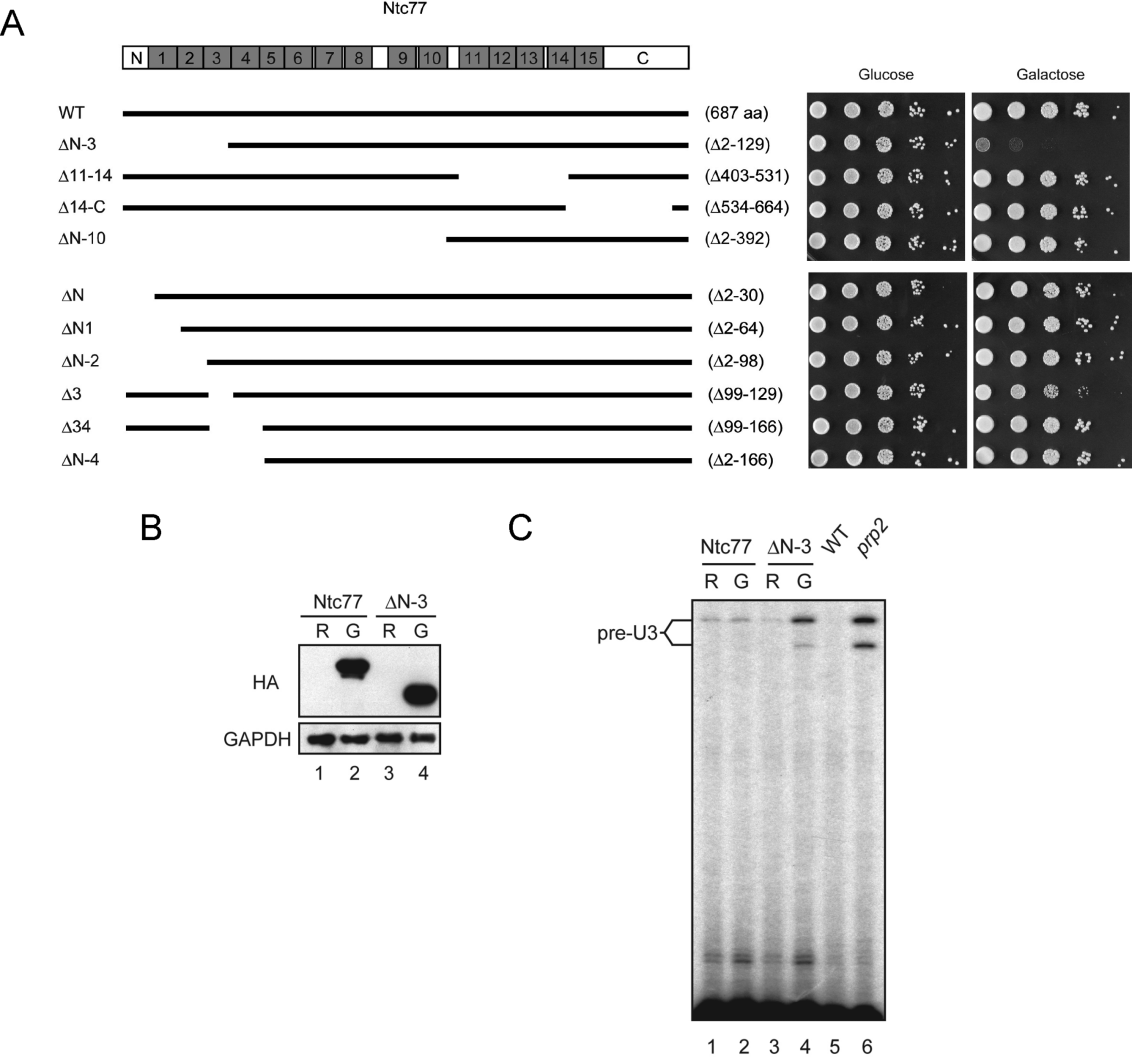
Overexpression of ΔN-3 of Ntc77 caused dominant-negative growth phenotype with a splicing defect. (**A**) Growth analysis for the overexpression of *NTC77* deletion mutants. Predicted TPR motifs of Ntc77 are shown in gray boxes. Black lines represent retained regions of the protein in deletion mutants. Cells harboring pRS426 plasmid containing *NTC77* deletion mutants under the control of *GAL1*-promoter were analyzed for growth in glucose- and galactose-based media by spot assays with 10-fold serial dilutions. (**B**) Western blotting of total proteins from lysates extracted from Ntc77- (lanes 1 and 2) or ΔN-3-overexpressed cells (lanes 3 and 4) after incubation in raffinose- (lanes 1 and 3) or galactose-based media (lanes 2 and 4). (**C**) Primer extension analysis. Total RNA extracted from Ntc77- or ΔN-3-overexpressed cells was analyzed by primer extension using a U3 primer R13. The *prp2-1* mutant was grown at 37°C for 2 h before harvest. WT, wild-type; R, raffinose; G, galactose.

Ntc77 contains 15 tandem TPR motifs flanked by short N-terminal and C-terminal domains (24). We deleted several TPR motifs in Ntc77 to seek for dominant-negative mutants of *NTC77* (Figure 2A). The deletion mutants of *NTC77* were placed under the control of *GAL1*-promoter on 2μ-based plasmid vector pRS426, and overexpression of the mutant proteins were induced by incubation in the presence of galactose. The result showed that overexpression of the mutant with deletion of the first 129 amino acid residues of Ntc77, encompassing NTD and the first three TPR elements (ΔN-3), caused a dominant-negative phenotype (Figure 2B). Western blotting confirmed expression of the ΔN-3 protein when grown in galactose-containing media (Figure 2B, lane 4). The ΔN-3 mutant also conferred splicing defect as revealed by primer extension analysis using U3 as a probe (Figure 2C, lane 4).

To further define the region whose deletion would cause the dominant-negative phenotype, we constructed several smaller deletions in the region, as well as one extended to the fourth TPR. Interestingly, deletion of only the third TPR (Δ3) displayed a mild dominant-negative phenotype and deletion of the N-terminus up to the second TPR (ΔN-2) gave no phenotype, suggesting that the third TPR might be the key component determining the phenotype. Further deletion of the fourth TPR, in ΔN-4 or Δ34, resulted in loss of dominant-negative phenotype, possibly due to destabilization of the proteins as the levels of the proteins were much lower than that of the wild-type or ΔN-3 (Supplementary Figure S2).

**Figure 3.**
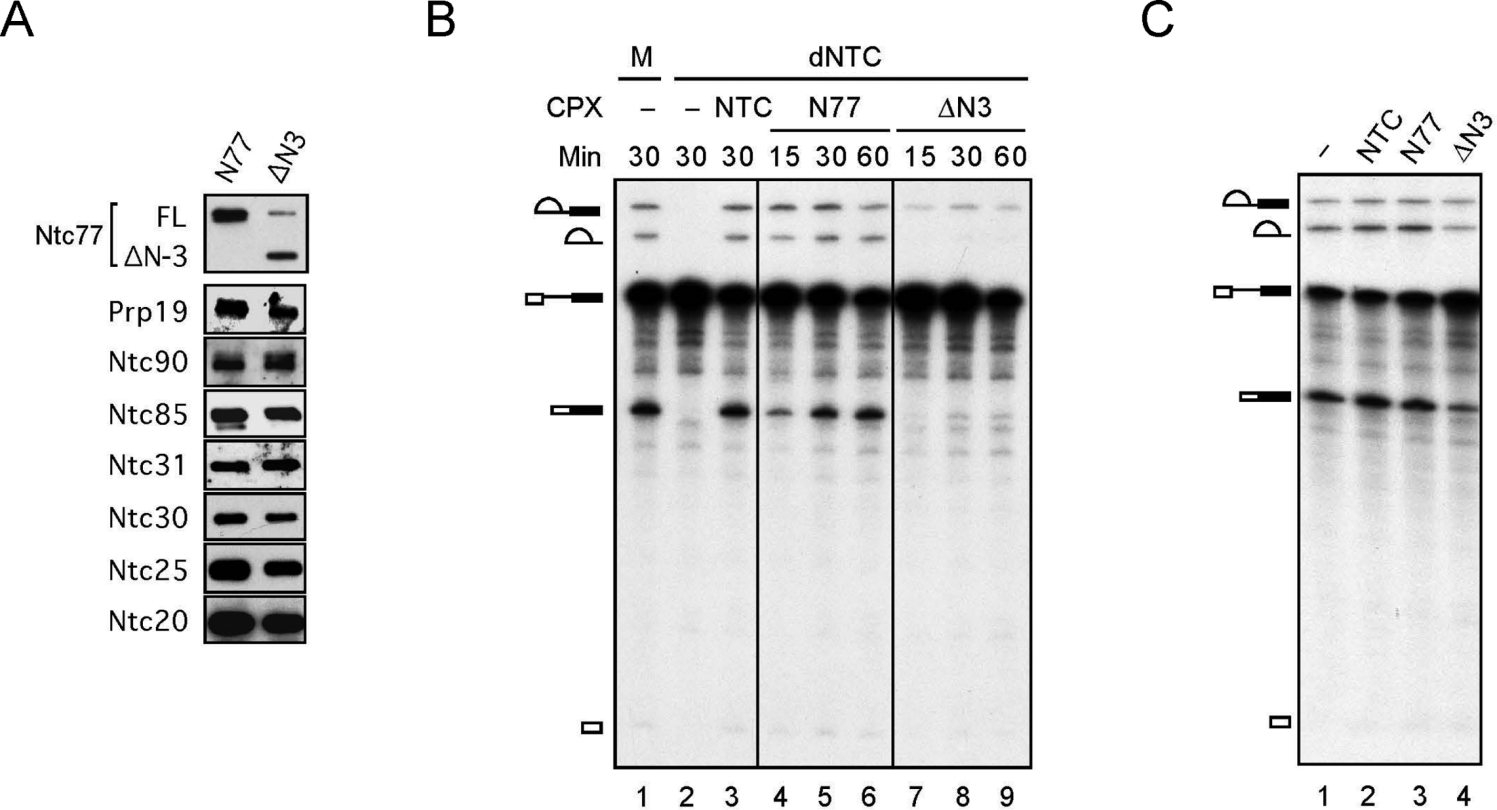
*In vitro* analysis of NTC containing full-length or ΔN-3 of Ntc77. (**A**) Western blotting of N77 and ΔN3 probed with antibodies against HA-epitope for Ntc77 and components of NTC. A weak band migrating near full-length Ntc77 in ΔN3 is the background from the anti-HA antibody. (**B**) Splicing reactions were carried out for 15, 30 or 60 min in mock- (lane 1) or Prp19-depleted extracts (lanes 2–9) without (lanes 1 and 2) or with the addition of NTC (lane 3), N77 (lanes 4–6) or ΔN3 (lanes 7–9). (**C**) Splicing reactions were carried out in wild-type extracts without (lane 1) or with the addition of NTC (lane 2), N77 (lane 3) or ΔN3 (lane 4). FL, full-length Ntc77; M, mock-depleted extracts; dNTC, Prp19-depleted extracts; CPX, complex.

To understand the functional effect of ΔN-3, we first examined whether the NTC is intact in the ΔN-3 mutant. A yeast strain expressing the HA-tagged ΔN-3 of Ntc77 with wild-type *NTC77* expression regulated by *GAL1*-promoter was used for preparation of splicing extracts after growth of cells in glucose-based medium for 20 h (Supplementary Figure S3A). The extract gave only residual amounts of splicing activity, and the activity could be partially rescued upon the addition of purified NTC (Supplementary Figure S3B). The complexes associated with full-length and ΔN-3 HA-tagged Ntc77 were purified by chromatography on anti-HA antibody columns from these extracts (named N77 for full-length, and ΔN3 for ΔN-3 of Ntc77-associated complexes). Western blotting of the N77 and ΔN3 components revealed no substantial difference in the amounts of NTC components co-purified with full-length or ΔN-3 of Ntc77 (Figure 3A), indicating that the integrity of NTC is not affected by deletion of NTD and the first three TPR elements. Assays for complementation of NTC-depleted extracts (Figure 3B) revealed that while the N77 complex rescued full splicing activity as NTC, (lanes 4–6), the ΔN3 complex barely rescued the splicing activity (lanes 7–9). Judging from the fact that the integrity of NTC is not affected but some function prior to or for the first catalytic reaction is impaired in ΔN3 complex, we speculated that the ΔN3 complex might be able to bind to the spliceosome. We therefore tested whether the ΔN3 complex could recapitulate the dominant-negative growth phenotype *in vitro* by adding excessive amounts of the purified ΔN3 complex to the splicing extract. Indeed, while the addition of NTC or N77 slightly enhanced the splicing activity of the extract (Figure 3C, lanes 1–3), the addition of the ΔN3 complex reduced the splicing activity by around 50% (lane 4), indicative of a dominant-negative phenotype *in vitro*.

**Figure 4.**
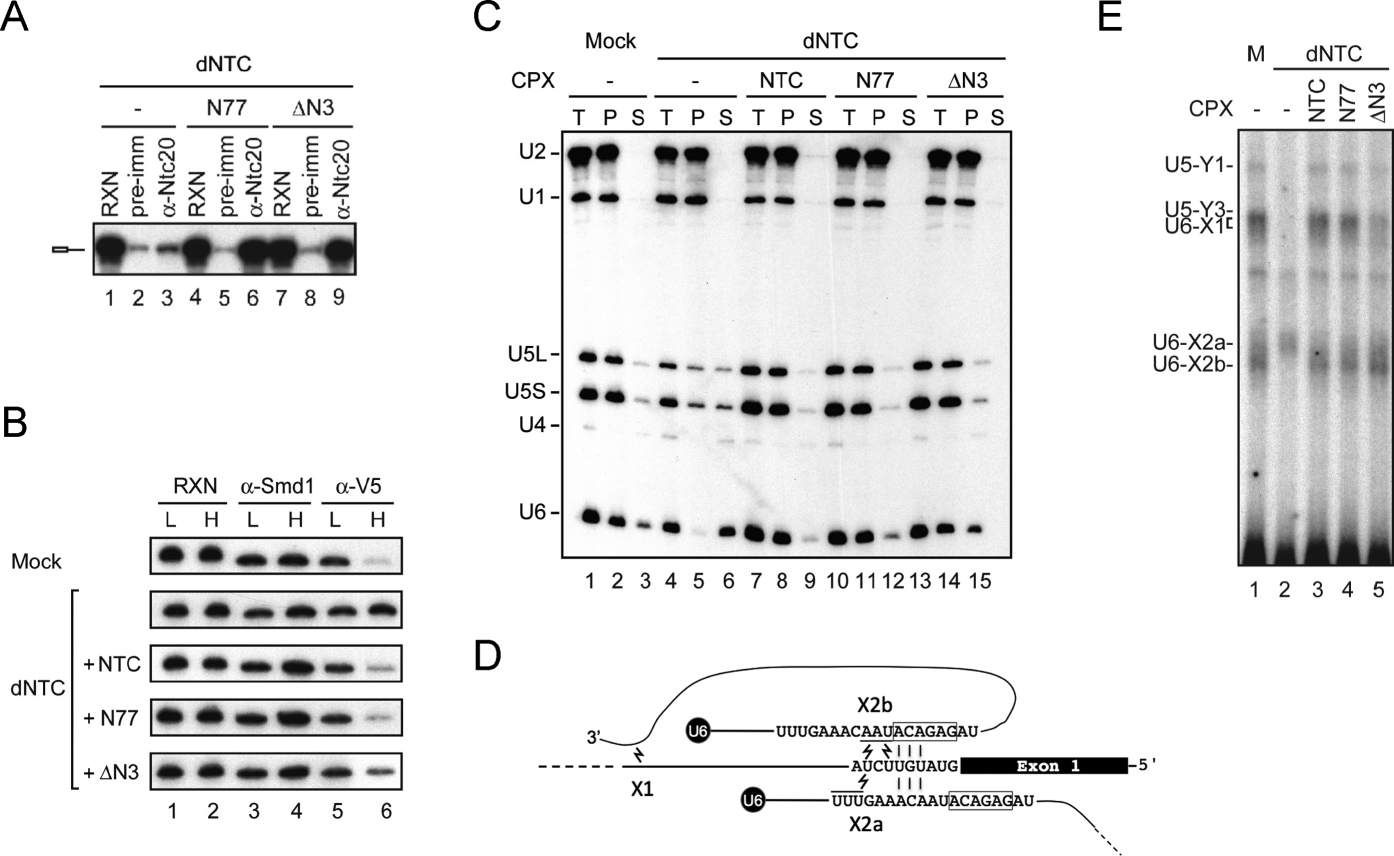
Spliceosome activation was not affected by deletion of the N-3 region of Ntc77. (**A**) Splicing reactions were carried out in Prp19-depleted extracts supplemented without (lanes 1–3) or with N77 (lanes 4–6) or ΔN3 (lanes 7–9), and the reaction mixtures were precipitated with pre-immune serum (lanes 2, 5 and 8) or anti-Ntc20 antibody (lanes 3, 6 and 9). (**B**) Splicing reactions were carried out using Ac/Cla pre-mRNA in the presence of 0.05 mM (lanes 1, 3 and 5) or 2 mM (lanes 2, 4 and 6) ATP, in mock- (top panel) or Prp19-depleted Lsm-V5 extracts (lower panels) supplemented without (second panel) or with NTC (third panel), N77 (fourth panel) or ΔN3 (bottom panel). The reaction mixtures were immunoprecipitated with anti-Smd1 (lanes 3 and 4) or anti-V5 antibody (lanes 5 and 6). (**C**) Splicing reactions were carried out with biotinylated ACAC pre-mRNA in mock- (lanes 1–3) or Prp19-depleted extracts (lanes 4–15) supplemented without (lanes 1–6) or with NTC (lanes 7–9), N77 (lanes 10–12) or ΔN3 (lanes 13–15). The spliceosomes precipitated with streptavidin Sepharose were incubated in splicing buffer containing ATP at 25°C for 20 min, and pellet and supernatant fractions were separated and then analyzed by northern blotting. **(D)** Schematic of U6-pre-mRNA interactions and products of UV-crosslinking. (**E**) Splicing reactions were carried out as in **(C)** but using ^32^P-labeled Ac/Cla pre-mRNA, and the spliceosomes were immunoprecipitated with anti-Smd1 antibody. After irradiation with UV_254 nm_, RNA was extracted and fractionated on a 5% polyacrylamide gel. RXN, 1/10 of reaction mix; pre-imm, pre-immune serum; dNTC, Prp19-depleted extracts; L, low ATP concentration; H, high ATP concentration; CPX, complex; T, total precipitates; P, pellet; S, supernatant.

To confirm that the ΔN3 complex is able to bind to the spliceosome, we analyzed the association of NTC with the spliceosome by immunoprecipitation with anti-Ntc20 antibody using a 3′-truncated actin pre-mRNA Ac/Cla, which retains only five bases downstream from the branchpoint (34). We have previously shown that the spliceosome can assemble on the Ac/Cla pre-mRNA up to the post-activation step, allowing binding of NTC and Prp2 but preventing the adenosine triphosphate (ATP)-dependent function of Prp2 in displacing SF3a/b (35). The result showed that the anti-Ntc20 antibody could precipitate the spliceosome formed in extracts containing N77 or ΔN3 complex (Figure 4A, lanes 6 and 9), indicating that deletion of the N-3 region of Ntc77 did not affect the binding of NTC to the spliceosome.

We then examined whether ΔN-3 is functional for mediating spliceosome activation.

We have previously demonstrated several characteristics of NTC-mediated spliceosome activation, including stabilization of U5 and U6 on the spliceosome, destabilization of Lsm from U6 and interactions of the Lsm binding site of U6 with the intron sequence around 30 nt downstream of the 5′ splice site (6). We therefore assessed whether ΔN-3 is functional for mediating spliceosome activation by examining these features. To see whether Lsm is destabilized from U6, splicing reactions were carried out using Ac/Cla pre-mRNA in NTC-depleted Lsm3-V5 extracts complemented with purified NTC, N77 or ΔN3 complex in the presence of low or high concentration of ATP, for pre- and post-activated spliceosomes, respectively. The reaction mixtures were subjected to immunoprecipitation with antibodies against Smd1 and V5 (Figure 4B). As shown before, Lsm3 accumulated on the spliceosome at high ATP concentrations in NTC-depleted extracts (lane 6 of second panel). When the purified NTC (third panel) or N77 complex (fourth panel) was added, only around 20% of the spliceosome co-precipitated with Smd1 was co-precipitated with Lsm3 at high ATP concentrations (compare lane 6 with lane 4 in third and fourth panels), indicating destabilization of Lsm from U6. When the ΔN3 complex was added, around 40% of the spliceosome was co-precipitated with Lsm3 (compare lane 6 with lane 4 in the bottom panel). Quantification of the experiment is shown in Supplementary Figure S4. This suggests that the ΔN3 complex is functional in mediating spliceosome activation but with a slightly lower efficiency.

To examine for the ability of the ΔN3 complex in stabilizing U5 and U6 after the release of U1 and U4, splicing reactions were carried out as in Figure 4B using biotinylated ACAC pre-mRNA. The spliceosome was pulled down with streptavidin Sepharose, and incubated in the splicing buffer containing ATP. After incubation, the supernatant and pellet fractions were examined for snRNAs by northern blotting (Figure 4C). With mock-depleted extracts, U5 and U6 remained stably associated with the spliceosome (lanes 2 and 3), while about half of U5 and nearly all of U6 were released from the spliceosome formed in NTC-depleted extracts (lanes 5 and 6). The addition of purified NTC or N77 stabilized U5 and U6 (lanes 8, 9, 11 and 12). The ΔN3 complex showed a similar pattern to the N77 complex, although had slightly more U5 and U6 in the supernatant (lanes 14 and 15), indicating that ΔN3 remains functional in stabilizing the association of U5 and U6 with the spliceosome.

We have also shown that NTC is required for promoting specific interactions of U5 and U6 with the pre-mRNA as can be revealed by UV-crosslinking (6). Two major crosslinking products, X1 and X2, between U6 and pre-mRNA were observed. X1 is the product of crosslink between the Lsm binding site of U6 snRNA and the intron sequence around 30 bases downstream of the 5′ splice site, and X2 is crosslink of pre-mRNA at near the 5′ splice site with U6 in a region immediately upstream of the conserved ACAGAG-box (Figure 4D). When splicing was carried out in the absence of NTC, X2 migrated slightly slower and X1 was absent (6). The slower and faster migrating species of X2, named X2a and X2b, respectively (Figure 4E, lanes 1 and 3 for X2b, and lane 2 for X2a), are crosslinks of different residues of U6 to pre-mRNA due to a 5-nt shift of base pair interactions between U6 and the 5′ splice site. The crosslinking pattern with ΔN3 (lane 5) was similar to that of NTC (lane 3), N77 (lane 4) or mock-treated extracts (lane 1), except with a slightly lower amount of X1 and a slightly greater amount of X2a. Two major and two minor crosslinking products between U5 and the 5′ splice site were also detected. The major crosslinks, Y1 and Y3, were much weaker in the absence of NTC (lane 2) (6), and were recovered upon the addition of NTC, N77 or ΔN3, but not as well with ΔN3. Taken together, these results show that deletion of the N-terminus of Ntc77 up to the third TPR does not severely impact spliceosome activation.

### The N-3 region of Ntc77 is not required for the Prp2 functioning step

**Figure 5.**
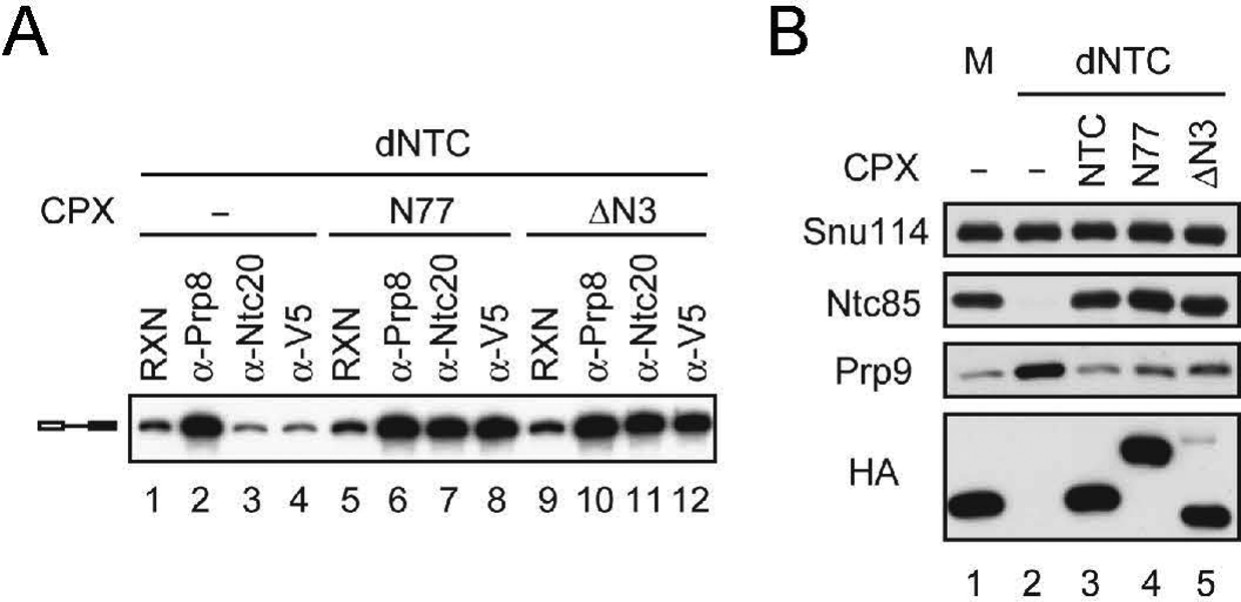
ΔN-3 does not affect the binding or action of Prp2. (**A**) Splicing reactions were carried out in Prp19-depleted *prp2-1* mutant extracts supplemented with recombinant 4V5-Prp2-S378L protein without (lanes 1–4) or with the addition of N77 (lands 5–8) or ΔN3 (lanes 9–12) and immunoprecipitated with anti-Prp8 (lanes 2, 6 and 10), anti-Ntc20 (lanes 3, 7 and 11) or anti-V5 antibody (lanes 4, 8 and 12). (**B**) Splicing reactions were carried out in mock- (lane 1) or Prp19-depleted extracts (lanes 2–5) without (lanes 1 and 2) or supplemented with NTC (lane 3), N77 (lane 4) or ΔN3 (lane 5) using biotinylated ACAC pre-mRNA, and the spliceosomes were pulled down with streptavidin Sepharose. Components were probed with antibodies against Snu114, Ntc85, Prp9 and HA-epitope for Prp19 (lanes 1 and 3) and Ntc77 (lanes 4 and 5). M, mock-depleted extracts; dNTC, Prp19-depleted extracts; RXN, 1/10 of reaction mix; CPX, complex; N77, Ntc77-associated complex; ΔN3, ΔN-3-associated complex.

After activation of the spliceosome, several proteins are required to promote the first catalytic reaction. Prp2 is first required to destabilize U2 components SF3a/b from binding to the branch site in an ATP-dependent manner (7,8), and its function requires Spp2 and Cwc22 (22,36). Yju2 and Cwc25 are then required to promote the first reaction in an ATP-independent manner. Since the N-terminal region of Ntc77 is not required for spliceosome activation, it may be required for the ATP-dependent step in supporting Prp2 function, or for the post-Prp2 step that requires Yju2 and Cwc25 to promote the catalytic reaction. We first examined whether deletion of the N-3 region of Ntc77 prevents Prp2 binding to the spliceosome (Figure 5A). To retain Prp2 on the spliceosome, we used a dominant-negative mutant of *PRP2*, S378L, which carries a mutation in the SAT motif (37). Splicing reactions were carried out in Prp19-depleted *prp2-1* mutant extracts supplemented with N77 or ΔN3, with the addition of recombinant V5-tagged Prp2-S378L, which allows Prp2 binding but not its release from the spliceosome. The reaction mixtures were precipitated with anti-V5 antibody to assay for the association of Prp2 (lanes 4, 8 and 12), with Prp8 and Ntc20 as controls to reveal the amount of total spliceosome. The result shows that indeed Prp2-S378L was able to bind the spliceosome in the presence of ΔN3 (lane 12). We then examined whether deletion of the N-3 region impedes the function of Prp2 in mediating destabilization of SF3a/b from the spliceosome by examining the association of SF3a component Prp9 with the spliceosome (Figure 5B). Splicing was carried out using biotinylated ACAC pre-mRNA in NTC-depleted extracts supplemented with NTC, N77 or ΔN3, and the spliceosome was pulled down with streptavidin Sepharose to examine for its protein content by western blotting. The result showed that Prp9 accumulated in higher amounts in NTC-depleted extracts (lane 2), but was maintained at a low level in mock-treated extracts (lane 1) or upon addition of NTC (lane 3) or N77 (lane 4). Addition of ΔN3 also resulted in loss of Prp9 (lane 5), suggesting that the Prp2 function in destabilizing SF3a/b was not severely impaired by deletion of the N-3 region of Ntc77 (lane 5).

### The N-terminal region of Ntc77 is required for stable association of Yju2 with the spliceosome

**Figure 6.**
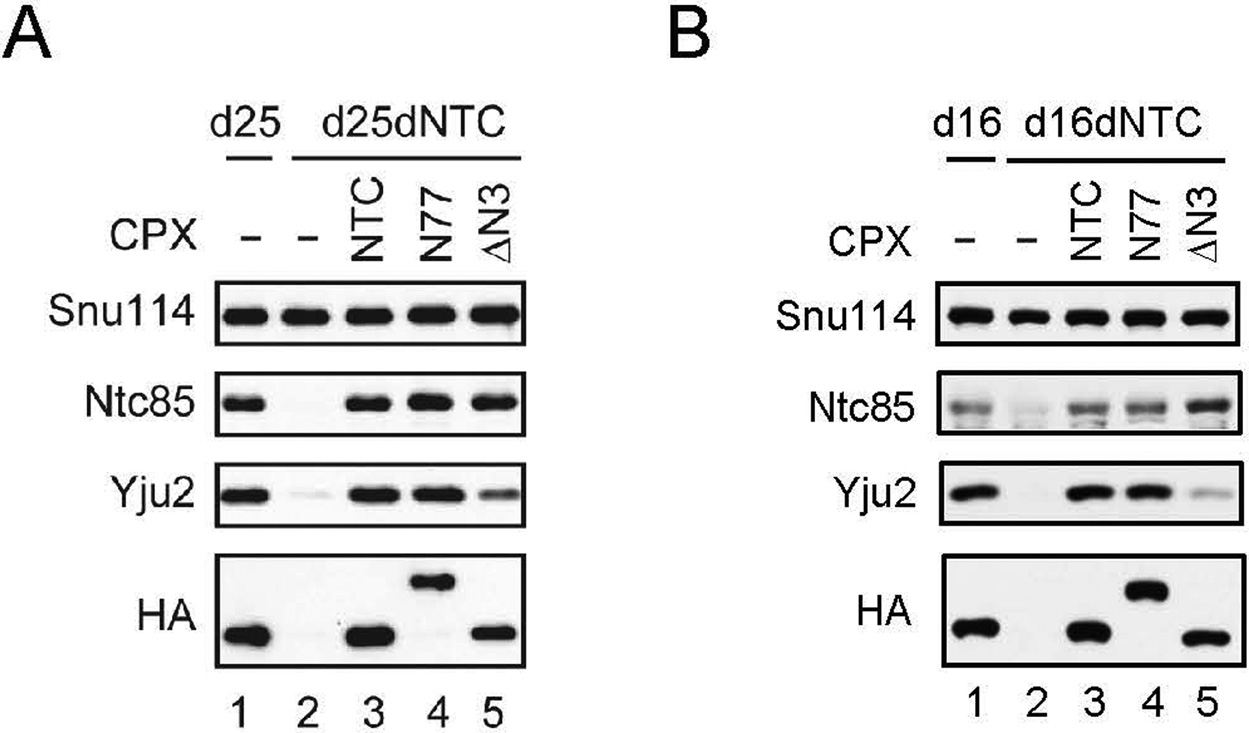
ΔN-3 destabilized the association of Yju2 with the spliceosome. (**A**) Splicing reactions were carried out in Cwc25- (lane 1) or Prp19- and Cwc25- doubly depleted extracts (lanes 2–5) without (lanes 1 and 2) or with the addition of NTC (lane 3), N77 (lane 4) or ΔN3 (lane 5) using biotinylated ACAC pre-mRNA, and the spliceosomes were pulled down with streptavidin Sepharose. CPX, complex; d25, Cwc25-depleted extracts; d25dNTC, Cwc25- and NTC-doubly depleted extracts. (**B**) Splicing reactions were carried out in Prp16- (lane 1) or Prp19- and Prp16-doubly depleted extracts (lanes 2–5) without (lanes 1 and 2) or with the addition of NTC (lane 3), N77 (lane 4) or ΔN3 (lane 5) using biotinylated ACAC pre-mRNA, and the spliceosomes were pulled down with streptavidin Sepharose. In both (A) and (B), components were probed with antibodies against Snu114, Ntc85, Yju2 and HA-epitope for Prp19 (lanes 1 and 3) and Ntc77 (lanes 4 and 5). CPX, complex; d16, Prp16-depleted extracts; d16dNTC, Prp16- and NTC-doubly depleted extracts.

We then examined whether the N-terminal region of Ntc77 is required for the function of Yju2 and Cwc25. Yju2 is required for the association of Cwc25 with the spliceosome to promote the first reaction, and can be recruited to the spliceosome before or after Prp2 action (10). We have previously shown that Ntc90 is not required for NTC-mediated spliceosome activation, and is required only for the recruitment of Yju2 to the spliceosome (28). Yju2 interacts with both Ntc90 and Ntc77, with Ntc90 via its NTD (Yju2-N) and with Ntc77 via its C-terminal domain (Yju2-C) (29). Yju2-C alone can bind stably to the spliceosome, but does not function in the first reaction. Furthermore, Yju2-C stabilizes the association of Yju2-N with the spliceosome to restore its full activity. Together, these results suggest that Ntc77 also plays a role in stabilization of Yju2. We speculated that the N-terminal region of Ntc77 might be involved in its interaction with Yju2, and examined the association of Yju2 with the spliceosome in ΔN-3. Splicing was carried out with biotinylated ACAC pre-mRNA in extracts depleted of both NTC and Cwc25, and supplemented with NTC, N77 or ΔN3. Depletion of Cwc25 results in the arrest of the spliceosome immediately before the first reaction, and accumulation of Yju2 on the spliceosome. The spliceosome was pulled down with streptavidin Sepharose to analyze the protein components (Figure 6A). Consistent with previous observation, Yju2 was barely detected in the absence of NTC (lane 2). While Yju2 was accumulated in a large amount on the spliceosome with N77 (lanes 3 and 4), less than 40% of Yju2 was present with ΔN3 (lane 5). The amount of Snu114 or Ntc85 was not significantly affected. This result indicates that the deletion of the N-3 region of Ntc77 might inhibit the splicing activity by preventing stable association of Yju2, and consequently the recruitment of Cwc25 to the spliceosome. After the first reaction, Yju2 and Cwc25 become stably associated with the spliceosome, and need to be displaced from the spliceosome mediated by Prp16. We further examined whether stable association of Yju2 with the spliceosome in post-catalytic stage is also affected by the presence of the N-3 region of Ntc77. Splicing was carried out with biotinylated ACAC pre-mRNA in extracts depleted of both NTC and Prp16, and supplemented with NTC, N77 or ΔN3 (Figure 6B). Similarly, less Yju2 was seen to be associated with the pulled-down ΔN3 spliceosome (lane 5), suggesting that the N-3 region of Ntc77 is required for stable association of Yju2 with both the pre- and post-catalytic spliceosome.

## DISCUSSION

Ntc77 was first identified as the yeast ortholog of *Drosophila* crooked neck protein, which is important for embryogenesis in *Drosophila* (24,38), and has been suggested to be involved in cell-cycle progression since loss of zygotic expression of *crn* gene results in the absence of specific neuronal lineage in embryonic CNS and PNS (38). *NTC77* was also identified as *SYF3* in the screening for synthetic lethal mutants in *prp17/cdc40Δ* cells (25), and depletion of Syf3 causes cell-cycle arrest (24). Mutations within several other splicing factors have been shown to result in cell-cycle arrest (38,39). It has been suggested that the cell-cycle phenotype may be ascribed to failure in removing introns from genes encoding factors involved in cell-cycle progression. The demonstration of rescue of cell-cycle arrest phenotype of *cef1–13* mutant upon removal of the *TUB1* intron supports this notion (40). It is thus possible that the cell-cycle phenotype of *NTC77* is a consequence of splicing defect.

Ntc77 contains 15 tandem copies of the TPR element. TPR-containing proteins were found in many protein complexes as a scaffold protein. The specificity of TPR is determined by the unconserved residues within the motif. Therefore, individual TPR elements within one polypeptide may play distinct functions by interacting with different partners. Supporting this notion, Vincent *et al.* showed that Ntc77 was differentially sensitive to different TPR deletions, and different TPR elements are not interchangeable (41). Consistently, we found that deletion of the third TPR element (Δ3) elicited more severe temperature-sensitive phenotype than deletion of the first TPR element (Δ1) (data not shown). Furthermore, overexpression of Ntc77Δ3 resulted in a mild dominant-negative phenotype. These results suggest that the third TPR element is important for the function of Ntc77.

Like Ntc90, Ntc77 interacts with five known NTC components, and with first-step factor Yju2 (17). Ntc77 and Ntc90 interact with distinct domains of Yju2, Ntc90 with Yju2-N and Ntc77 with Yju2-C (29). Yju2-N is partially functional for splicing with the second catalytic reaction more severely impeded than the first, and interacts with the spliceosome only weakly. Although Yju2-C is not essential for cellular growth, it binds stably to the spliceosome in the absence of Yju2-N, and greatly enhances the function of Yju2-N in splicing. Together, these results suggest that Ntc77 also plays a role in the first catalytic step by stabilizing the association of Yju2 with the spliceosome. In this work, we identified the N-terminal region of Ntc77, comprising the NTD and the first three TPR elements, to be important for stable association of Yju2 with the spliceosome. Deletion of N-3 did not significantly impact spliceosome activation, but destabilized Yju2 binding to the spliceosome. Since ΔN-3 also did not affect the association of NTC with the spliceosome, conceivably, the defective NTC complex is retained and stalled the spliceosome at the post-activation stage, which causes dominant-negative growth phenotype. Using the purified ΔN-3-containing complex, ΔN3, we were able to reproduce the dominant-negative feature of ΔN-3 in the *in vitro* splicing reaction. Since ΔN-3 only resulted in ∼60% reduction in the amount of Yju2 associated with the spliceosome, whether destabilization of Yju2 is the only cause of the first step defect is questionable. The fact that the presence of excessive amounts of recombinant Yju2 protein did not increase the splicing activity of ΔN-3 extracts (data not shown) suggests that additional factors might be involved. Besides interacting with Yju2, Ntc77 also interacts with another first step factor Cwc22 (data not shown). Nevertheless, ΔN-3 did not affect stable association of Cwc22 with the spliceosome (data not shown). Factors that may be responsible for the first step defect of ΔN-3 in combination with Yju2 destabilization remain to be uncovered.

The fact that the N-3 region of Ntc77 is not required for spliceosome activation, and the remaining portion of the protein can bind stably to the spliceosome to mediate spliceosome activation suggests that the N-3 region may constitute a distinct domain from the main body of the protein. It is interesting to note that antibodies raised against Ntc77 recognize full-length protein much better than the ΔN-3 protein. Furthermore, anti-Ntc77 antibodies inhibited the splicing reaction without affecting the integrity of the NTC complex (Supplementary Figure S5). Taken together, these results suggest that the antibody may primarily recognize the N-3 region of the protein, which may either form a distinct domain or assume a more disordered structure for interaction with Yju2-C.

Deletion of the N-3 region of Ntc77 resulted in destabilization, but not complete disruption, of the association of Yju2 with the spliceosome. We initially speculated that deletion of more sequence from the N-terminus of Ntc77 might be necessary to completely eliminate its function for interaction with Yju2. Nevertheless, further deletion into TPR-4 or deletion of only TRR-3 or TPR-4 abolished the dominant-negative growth phenotype. Immunoprecipitation analysis revealed that ΔN-4 did not greatly affect the integrity of NTC, but the binding of NTC to the spliceosome was slightly impaired (data not shown). Moreover, western blot analysis of splicing extracts revealed much lower levels of Ntc77 protein in ΔN-4 and Δ34 than in wild-type or ΔN-3 extracts (Supplementary Figure S2 and data not shown), indicating that deletion of TPR-4 might result in instability of the protein. These results suggest that the lack of dominant-negative phenotype of ΔN-4 might be primarily attributed to insufficient levels of ΔN-4. It is possible that further deletion of the N-terminal region may disrupt the function of Ntc77 in spliceosome activation. Although the N-3 region of Ntc77 is important for stabilizing the association of Yju2 with the spliceosome, the remaining portion of the protein may also contribute to its interaction with Yju2.

In a large-scale screening of two-hybrid interactions, an N-terminal segment of Ntc77, containing amino acid residues 11–156, was found to interact with Ntc85 using Ntc85 as bait (25). We found that deletion of this region in ΔN-3 or ΔN-4 did not affect the association of Ntc77 with Ntc85 by immunoprecipitation analysis (Figure 1 for ΔN-3, and data not shown for ΔN-4). We also observed two-hybrid interaction between Ntc85 and ΔN-3 (data not shown). These results suggest that Ntc85 might interact with multiple regions of Ntc77, and the interaction in the N-terminus may not be important for formation of NTC.

Ntc77 has previously been suggested to mediate the transition from the pre-spliceosome to the pre-activated spliceosome by recruiting U4/U6.U5 tri-snRNP (24). However, we demonstrated that Ntc77 is a component of the NTC and associates with the spliceosome in the same manner as Prp19 (18). We further show here that in the absence of Ntc77, the interaction between Ntc85 and Ntc90 is disrupted (Figure 1B), which likely affects the integrity of NTC since the binding of NTC components to the spliceosome is inhibited (Figure 1C). NTC is not required for the release of U1 and U4 from the spliceosome, and is required for stable association of U5 and U6 with the spliceosome after the release of U1 and U4 during spliceosome activation (6). We have shown that spliceosomes assembled in the absence of NTC consistently contain lower amounts of U5 and U6 than in the presence of NTC, and upon incubation of the purified spliceosome in the splicing buffer, U5 and U6 are further dissociated from the spliceosome (6). Weak association of U5 and U6 with the spliceosome could result in retention of only very small amounts of U5 and U6 on the purified spliceosome, and could be easily interpreted as failure in recruiting the tri-snRNP to the spliceosome. A recent report on essential splicing factor Sad1 has revealed a similar scenario. Sad1 was initially identified in a screen for mutations defective in snRNP assembly (42), and has recently been shown to promote formation of U4/U6.U5 tri-snRNP, counteracting Brr2-mediated, ATP-dependent tri-snRNP dissociation in tri-snRNP homeostasis (43). Depletion of Sad1 from the extract results in no binding of the tri-snRNP under normal splicing conditions due to the lack of functional tri-snRNP, but has no effect on tri-snRNP binding when splicing is carried out at lower concentrations of ATP. We have indeed also observed binding of the tri-snRNP to the spliceosome in Ntc77-depleted extracts when splicing was carried out at lower ATP concentrations (data not shown), arguing against a role of Ntc77 in promoting tri-snRNP binding to the spliceosome.

## SUPPLEMENTARY DATA

Supplementary Data are available at NAR Online.

SUPPLEMENTARY DATA

## References

[1] Wahl M.C., Will C.L., Lührmann R.L. (2009). The spliceosome: design principles of a dynamic RNP machine. Cell.

[2] Raghunathan P.L., Guthrie C. (1998). RNA unwinding in U4/U6 snRNPs requires ATP hydrolysis and the DEIH-box splicing factor Brr2. Curr. Biol..

[3] Staley J.P., Guthrie C. (1999). An RNA switch at the 5′ splice site requires ATP and the DEAD box protein Prp28p. Mol. Cell.

[4] Chen J.Y.-F., Stands L., Staley J.P., Jackups R.R., Latus L.J., Chang T.-H. (2001). Specific alterations of U1-C protein or U1 small nuclear RNA can eliminate the requirement of Prp28p, an essential DEAD box splicing factor. Mol. Cell.

[5] Chan S.-P., Cheng S.-C. (2005). The Prp19-associated complex is required for specifying interactions of U5 and U6 with Pre-mRNA during spliceosome activation. J. Biol. Chem..

[6] Chan S.-P., Kao D.-I., Tsai W.-Y., Cheng S.-C. (2003). The Prp19p-associated complex in spliceosome activation. Science.

[7] Lardelli R.M., Thompson J.X., Yates J.R., Stevens S.W. (2010). Release of SF3 from the intron branchpoint activates the first step of pre-mRNA splicing. RNA.

[8] Warkocki Z., Odenwalder P., Schmitzova J., Platzmann F., Stark H., Urlaub H., Ficner R., Fabrizio P., Lührmann R. (2009). Reconstitution of both steps of Saccharomyces cerevisiae splicing with purified spliceosomal components. Nat. Struct. Mol. Biol..

[9] Chiu Y.-F., Liu Y.-C., Chiang T.-W., Yeh T.-C., Tseng C.-K., Wu N.-Y., Cheng S.-C. (2009). Cwc25 is a novel splicing factor required after Prp2 and Yju2 to facilitate the first catalytic reaction. Mol. Cell. Biol..

[10] Liu Y.-C., Chen H.-C., Wu N.-Y., Cheng S.-C. (2007). A novel splicing factor Yju2 is associated with NTC and acts after Prp2 in promoting the first catalytic reaction of pre-mRNA splicing. Mol. Cell. Biol..

[11] Tseng C.-K., Liu H.-L., Cheng S.-C. (2011). DEAH-box ATPase Prp16 has dual roles in remodeling of the spliceosome in catalytic steps. RNA.

[12] Schwer B., Gross C.H. (1998). Prp22, a DExH-box RNA helicase, plays two distinct roles in yeast pre-mRNA splicing. EMBO J..

[13] Ansari A., Schwer B. (1995). SLU7 and a novel activity, SSF1, act during the PRP16-dependent step of yeast pre-mRNA splicing. EMBO J..

[14] Horowitz D.S., Abelson J. (1993). Stages in the second reaction of pre-mRNA splicing: the final step is ATP independent. Genes Dev..

[15] Tsai W.-Y., Chow Y.-T., Chen H.-R., Huang K.-T., Hong R.-I., Jan S.-P., Kuo N.-Y., Tsao T.Y., Chen C.-H., Cheng S.-C. (1999). Cef1p is a component of the Prp19p-asociated complex and essential for pre-mRNA splicing. J. Biol. Chem..

[16] Chen H.-R., Jan S.-P., Tsao T.Y., Sheu Y.-J., Banroques J., Cheng S.-C. (1998). Snt309p, a component of the Prp19p-associated complex that interacts with Prp19p and associates with the spliceosome simultaneously with or immediately after dissociation of U4 in the same manner as Prp19p. Mol. Cell. Biol..

[17] Chen C.-H., Tsai W.-Y., Chen H.-R., Wang C.-H., Cheng S.-C. (2001). Identification and characterization of two novel components of the Prp19p-associated complex, Ntc30p and Ntc20p. J. Biol. Chem..

[18] Chen C.-H., Yu W.-C., Tsao T.Y., Wang L.-Y., Chen H.-R., Lin J.-Y., Tsai W.-Y., Cheng S.-C. (2002). Functional and physical interactions between components of the Prp19p-associated complex. Nucleic Acids Res..

[19] Hogg R., McGrail J.C., O'Keefe R.T. (2010). The function of the NineTeen Complex (NTC) in regulating spliceosome conformations and fidelity during pre-mRNA splicing. Biochem. Soc. Trans..

[20] Ajuh P., Kuster B., Panov K., Zomerdijk J.C.B.M., Mann M., Lamond A.I. (2000). Functional analysis of the human CDC5L complex and identification of its components by mass spectrometry. EMBO J..

[21] Ohi M.D., Link A.J., Ren L., Jennings J.L., McDonald W.H., Gould K.L. (2002). Proteomics analysis reveals stable multiprotein complexes in both fission and budding yeasts containing Myb-related Cdc5p/Cef1p, novel pre-mRNA splicing factors, and snRNAs. Mol. Cell. Biol..

[22] Yeh T.-C., Liu H.-L., Chung C.-S., Wu N.-Y., Liu Y.-C., Cheng S.-C. (2011). Cwc22 is a novel splicing factor required for the function of Prp2 and for the spliceosome to escape from a futile pathway. Mol. Cell. Biol..

[23] Fabrizio P., Dannenberg J., Dube P., Kastner B., Stark H., Urlaub H., Lührmann R. (2009). The evolutionarily conserved core design of the catalytic activation step of the yeast spliceosome. Mol. Cell.

[24] Chung S., Mclean M.R., Rymond B.C. (1999). Yeast ortholog of the *Drosophila* crooked neck protein promotes spliceosome assembly through stable U4/U6.U5 snRNP addition. RNA.

[25] Ben-Yehuda S., Dix I., Russell C.S., McGarvey M., Beggs J.D., Kupiec M. (2000). Genetic and physical interactions between factors involved in both cell cycle progression and pre-mRNA splicing in *Saccharomyces cerevisiae*. Genetics.

[26] Lamb J.R., Tugendreich S., Hieter P. (1995). Tetratrico peptide repeat interactions: to TPR or not to TPR?. TIBS.

[27] D'Andrea L.D., Regan L. (2003). TPR proteins: the versatile helix. TIBS.

[28] Chang K.-J., Chen H.-C., Cheng S.-C. (2009). Ntc90 is required for recruiting first step factor Yju2 but not for spliceosome activation. RNA.

[29] Chiang T.-W., Cheng S.-C. (2013). A weak spliceosome-binding domain of Yju2 functions in first step and bypasses Prp16 in second step of splicing. Mol. Cell. Biol..

[30] Cheng S.-C., Newman A., Lin R.-J., McFarland G.D., Abelson J.N. (1990). Preparation and fractionation of yeast splicing extract. Meth. Enzymol..

[31] Tarn W.-Y., Hsu C.-H., Huang K.-T., Chen H.-R., Kao H.-Y., Lee K.-R., Cheng S.-C. (1994). Functional association of essential splicing factor(s) with PRP19 in a protein complex. EMBO J..

[32] Das A.K., Cohen P.W., Barford D. (1998). The structure of the tetratricopeptide repeats of protein phosphatase 5: implications for TPR-mediated protein-protein interactions. EMBO J..

[33] Chen H.-R., Tsao T.Y., Chen C.-H., Tsai W.-Y., Her L.-S., Hsu M.-T., Cheng S.-C. (1999). Snt309p modulates interactions of Prp19p with its associated components to stabilize the Prp19p-associated complex essential for pre-mRNA splicing. Proc. Natl. Acad. Sci. U.S.A..

[34] Vijayraghavan U., Parker R., Tamm J., Iimura Y., Rossi J., Abelson J., Gurthrie C. (1986). Mutations in conserved intron sequences affect multiple steps in the yeast splicing pathway, particularly assembly of the spliceosome. EMBO J..

[35] Cheng S.-C. (1994). Formation of the yeast splicing complex A1 and association of the splicing factor PRP19 with the pre-mRNA are independent of the 3′ region of the intron. Nucleic Acids Res..

[36] Liu H.-L., Cheng S.-C. (2012). The interaction of Prp2 with a defined Region of the intron is required for the first splicing reaction. Mol. Cell. Biol..

[37] Roy J., Kim K., Maddock J.R., Anthony J.G., Woolford J. Jr (1995). The final stages of spliceosome maturation require Spp2p that can interact with the DEAH box protein Prp2p and promote step 1 of splicing. RNA.

[38] Plumpton M., McGarvey M., Beggs J.D. (1994). A dominant negative mutation in the conserved RNA helicase motif ‘SAT’ causes splicing factor PRP2 to stall in spliceosomes. EMBO J..

[39] Zhang K., Smouse D., Perrimon N. (1991). The *crooked neck* gene of *Drosophila* contains a motif found in a family of yeast cell cycle genes. Genes Dev..

[40] Burns C.G., Ohi R., Mehta S., O'Toole E.T., Winey M., Clark T.A., Sugnet C.W., Ares M., Gould K.L. (2002). Removal of a single alpha-tubulin gene intron suppresses cell cycle arrest phenotypes of splicing factor mutations in Saccharomyces cerevisiae. Mol. Cell. Biol..

[41] Vincent K., Wang Q., Jay S., Hobbs K., Rymond B.C. (2003). Genetic interactions with CLF1 identify additional pre-mRNA splicing factors and a link between activators of yeast vesicular transport and splicing. Genetics.

[42] Lygerou Z., Christophides G., Séraphin B. (1999). A novel genetic screen for snRNP assembly factors in yeast identifies a conserved protein, Sad1p, also required for pre-mRNA splicing. Mol. Cell. Biol..

[43] Huang Y.-H., Chung C.-S., Kao D.-I., Kao T.-C., Cheng S.-C. (2014). Sad1 counteracts Brr2-mediated dissociation of U4/U6.U5 in tri-snRNP homeostasis. Mol. Cell. Biol..

