# Supplementary material for: Structural requirement of Ntc77 for spliceosome activation and first catalytic step
Source: Nucleic Acids Res. 2014 Oct 7;42(19):12261–71. doi: 10.1093/nar/gku914 (PMC4231770; doi:10.1093/nar/gku914)
Supplement: SUPPLEMENTARY DATA [file supp_42_19_12261__index.html]

Structural requirement of Ntc77 for spliceosome activation and first catalytic step — SUPPLEMENTARY DATA 

# Structural requirement of Ntc77 for spliceosome activation and first catalytic step

## SUPPLEMENTARY DATA

**Files in this Data Supplement:**

- SUPPLEMENTARY DATA
