# Supplementary material for: Structural requirement of Ntc77 for spliceosome activation and first catalytic step
Source: Nucleic Acids Res. 2014 Oct 7;42(19):12261–71. doi: 10.1093/nar/gku914 (PMC4231770; doi:10.1093/nar/gku914)
Supplement: SUPPLEMENTARY DATA [file supp_gku914_nar-02367-r-2014-File003.pptx]

## Slide 1
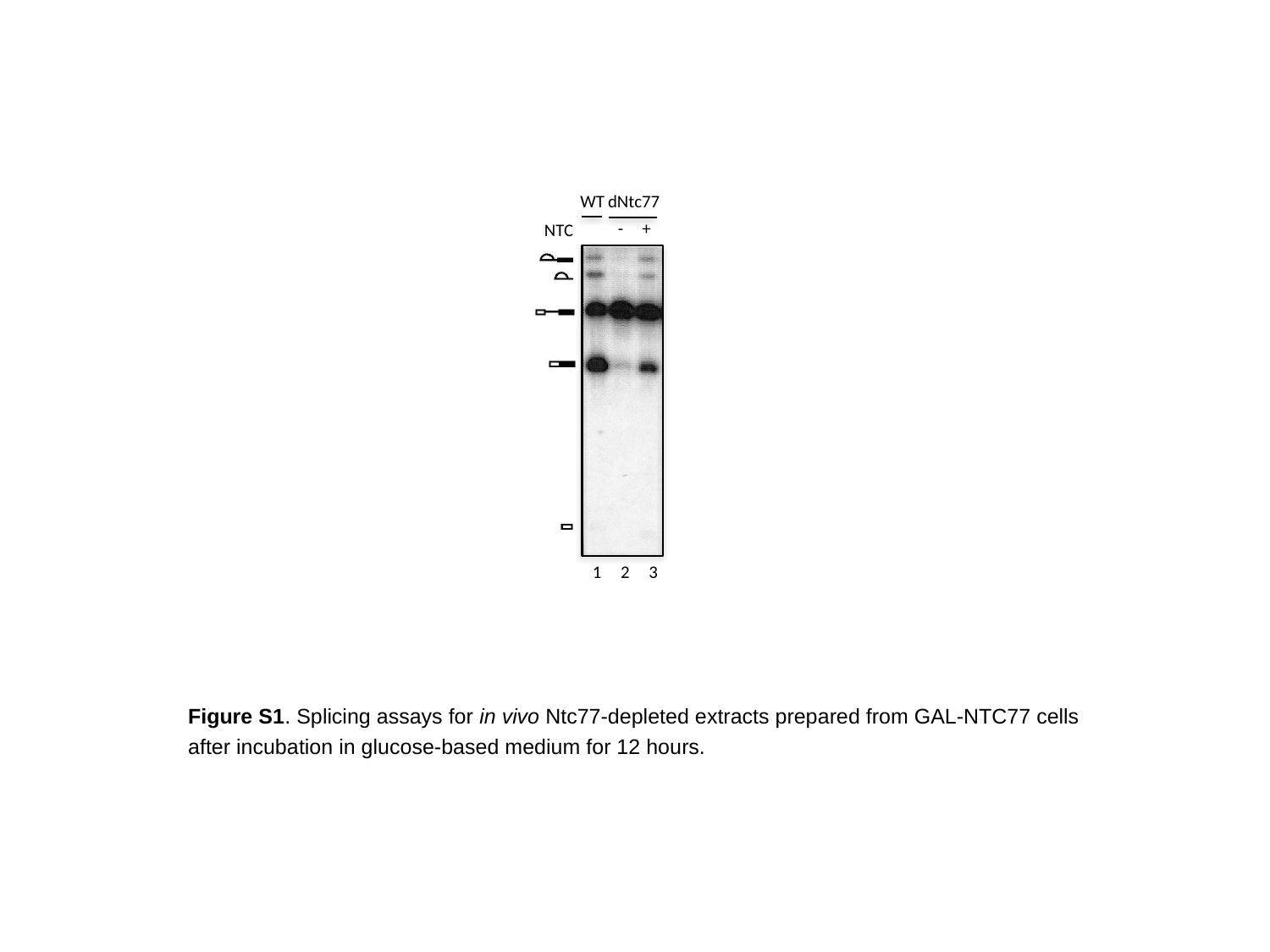

WT
dNtc77
-
+
NTC
1
2
3
Figure S1. Splicing assays for in vivo Ntc77-depleted extracts prepared from GAL-NTC77 cells after incubation in glucose-based medium for 12 hours.

## Slide 2
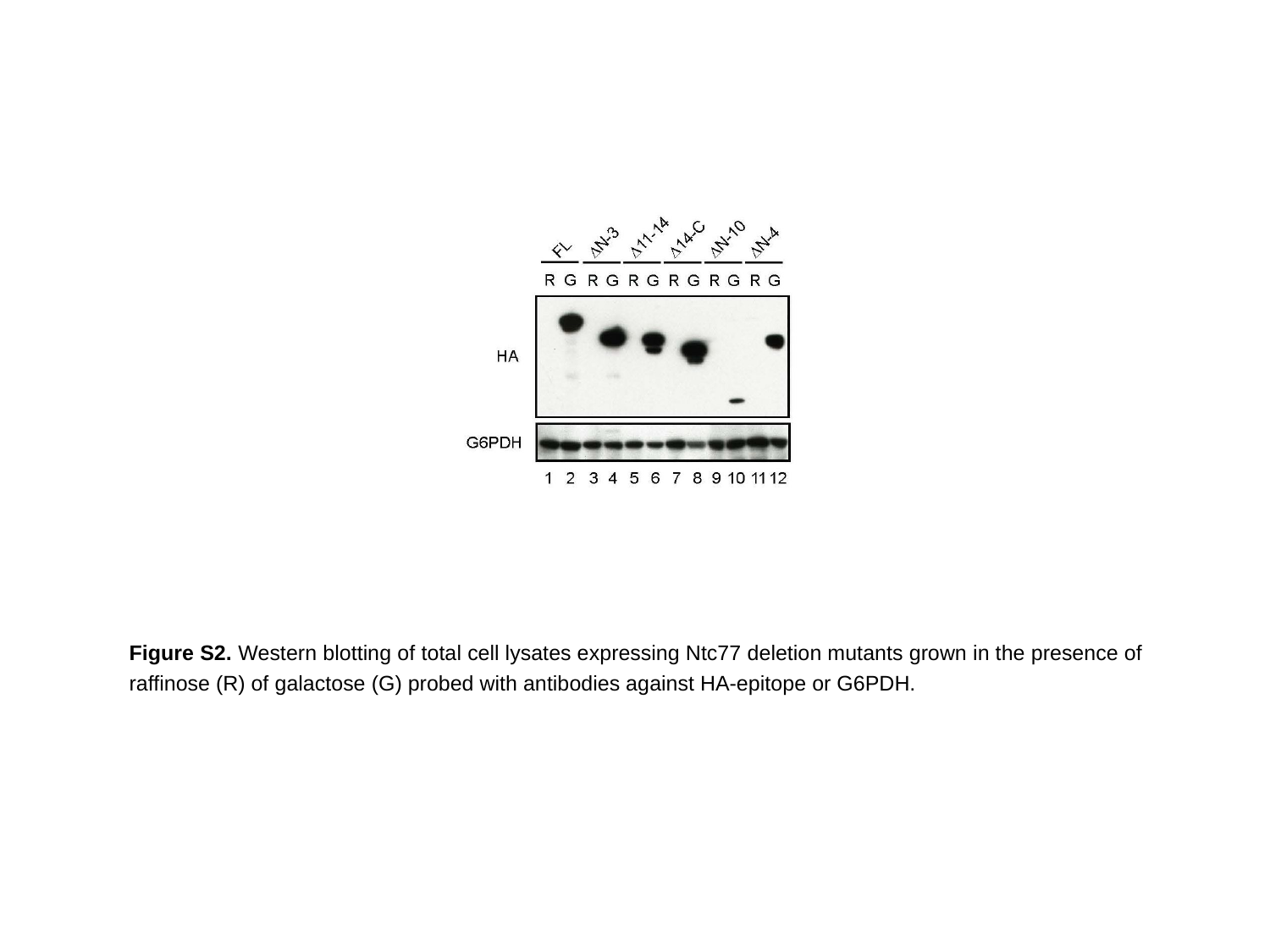

Figure S2. Western blotting of total cell lysates expressing Ntc77 deletion mutants grown in the presence of raffinose (R) of galactose (G) probed with antibodies against HA-epitope or G6PDH.

## Slide 3
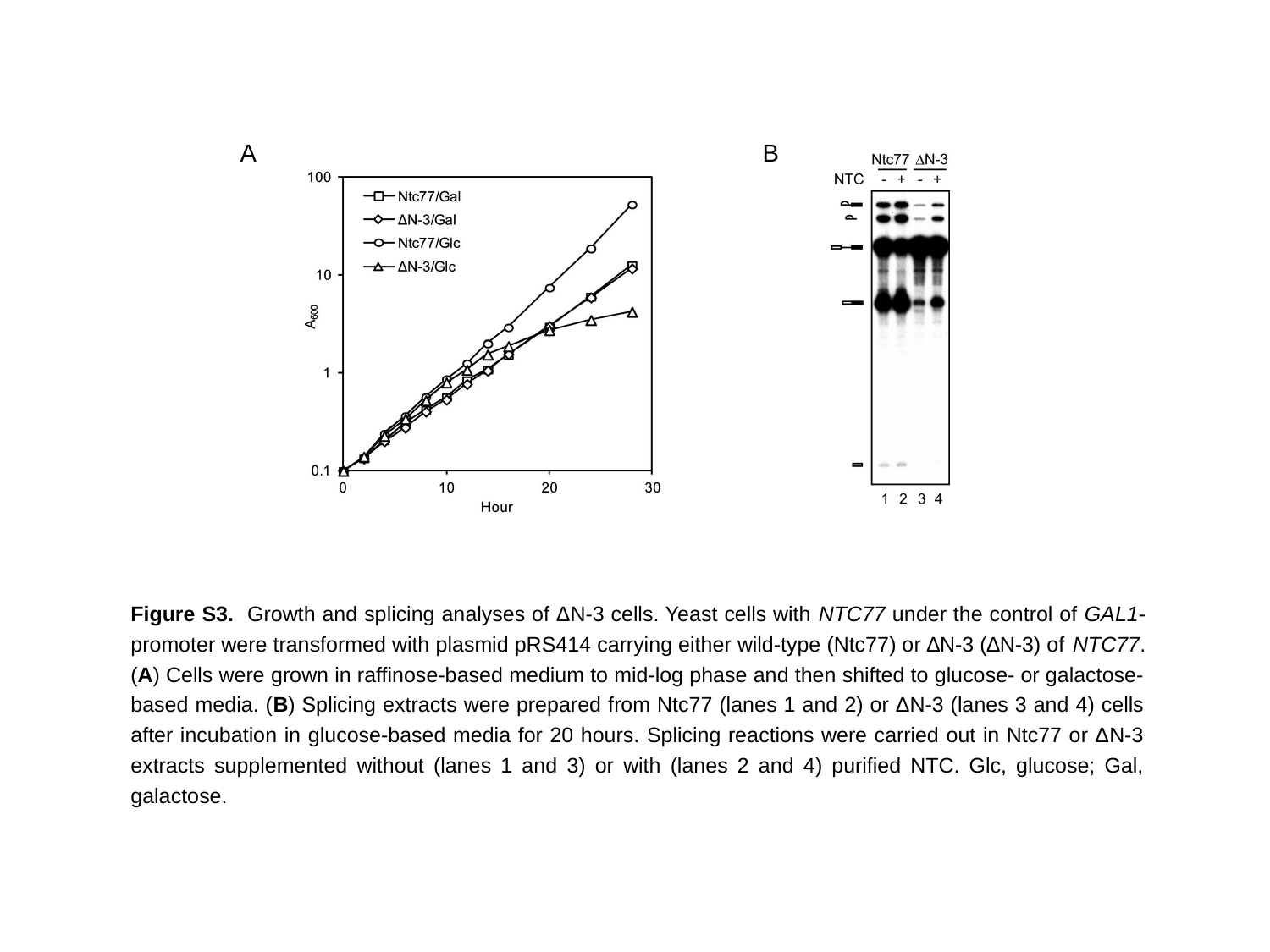

A
B
Figure S3. Growth and splicing analyses of ΔN-3 cells. Yeast cells with NTC77 under the control of GAL1-promoter were transformed with plasmid pRS414 carrying either wild-type (Ntc77) or ∆N-3 (∆N-3) of NTC77. (A) Cells were grown in raffinose-based medium to mid-log phase and then shifted to glucose- or galactose-based media. (B) Splicing extracts were prepared from Ntc77 (lanes 1 and 2) or ΔN-3 (lanes 3 and 4) cells after incubation in glucose-based media for 20 hours. Splicing reactions were carried out in Ntc77 or ΔN-3 extracts supplemented without (lanes 1 and 3) or with (lanes 2 and 4) purified NTC. Glc, glucose; Gal, galactose.

## Slide 4
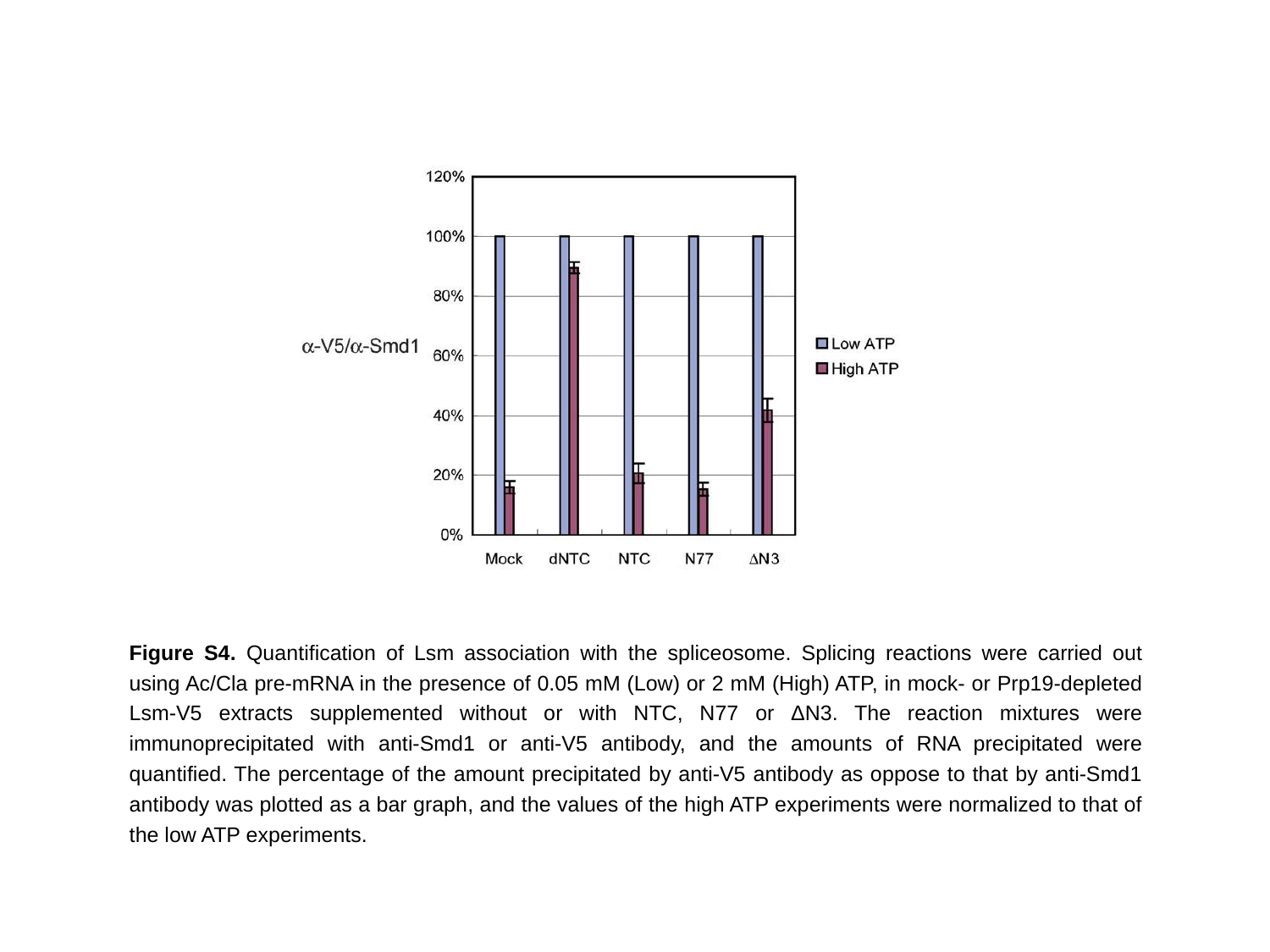

Figure S4. Quantification of Lsm association with the spliceosome. Splicing reactions were carried out using Ac/Cla pre-mRNA in the presence of 0.05 mM (Low) or 2 mM (High) ATP, in mock- or Prp19-depleted Lsm-V5 extracts supplemented without or with NTC, N77 or ΔN3. The reaction mixtures were immunoprecipitated with anti-Smd1 or anti-V5 antibody, and the amounts of RNA precipitated were quantified. The percentage of the amount precipitated by anti-V5 antibody as oppose to that by anti-Smd1 antibody was plotted as a bar graph, and the values of the high ATP experiments were normalized to that of the low ATP experiments.

## Slide 5
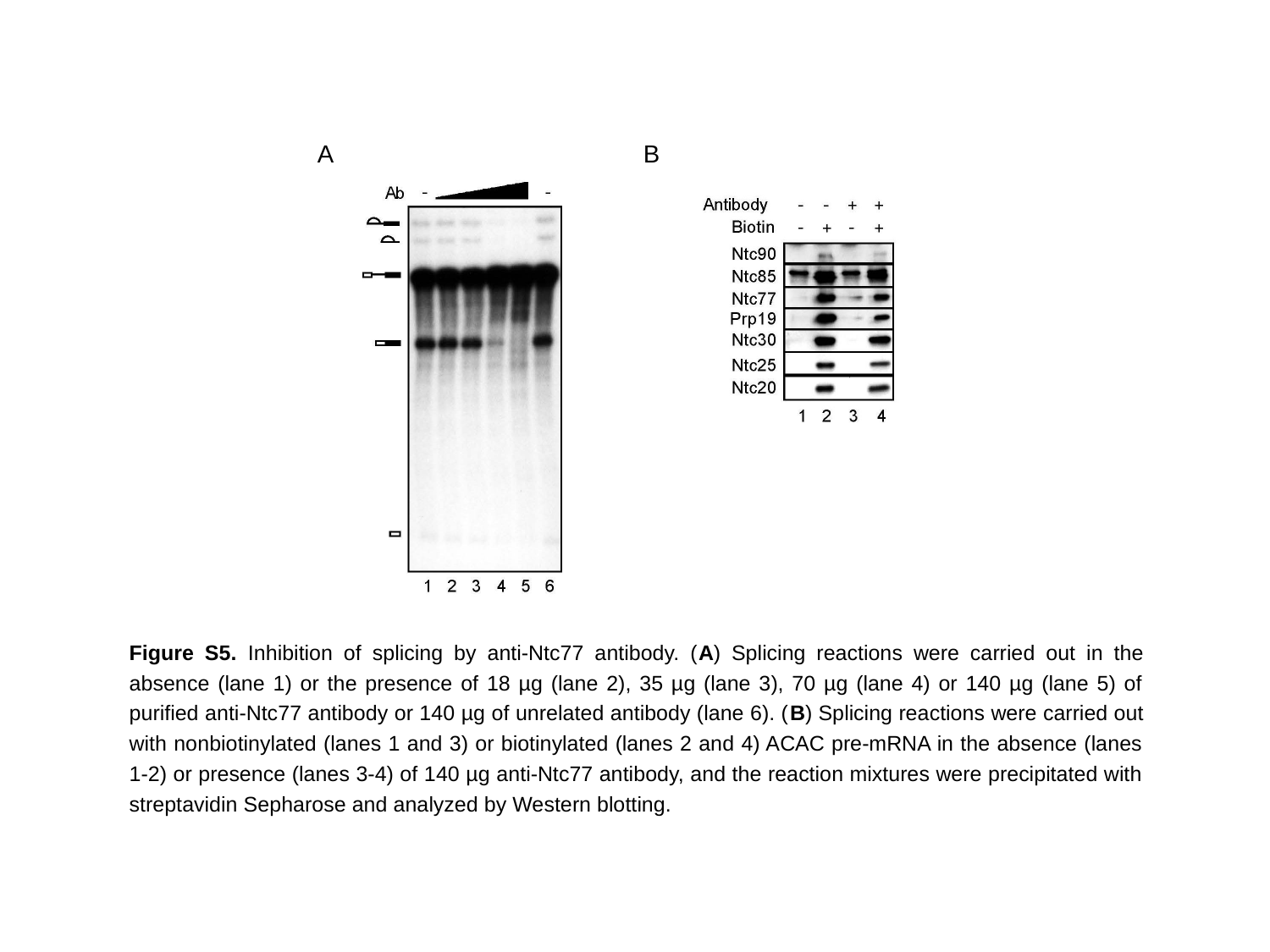

A
B
Figure S5. Inhibition of splicing by anti-Ntc77 antibody. (A) Splicing reactions were carried out in the absence (lane 1) or the presence of 18 µg (lane 2), 35 µg (lane 3), 70 µg (lane 4) or 140 µg (lane 5) of purified anti-Ntc77 antibody or 140 µg of unrelated antibody (lane 6). (B) Splicing reactions were carried out with nonbiotinylated (lanes 1 and 3) or biotinylated (lanes 2 and 4) ACAC pre-mRNA in the absence (lanes 1-2) or presence (lanes 3-4) of 140 µg anti-Ntc77 antibody, and the reaction mixtures were precipitated with streptavidin Sepharose and analyzed by Western blotting.
